# Supplementary material for: Vapor sublimation and deposition to build porous particles and composites
Source: Nat Commun. 2018 Jul 2;9:2564. doi: 10.1038/s41467-018-04975-2 (PMC6028631; doi:10.1038/s41467-018-04975-2)
Supplement: Supplementary file 1 — Supplementary Information [file 41467_2018_4975_MOESM1_ESM.pdf]

Supplementary Information

Vapor Sublimation and Deposition to Build Porous Particles and Composites

Tung et al.

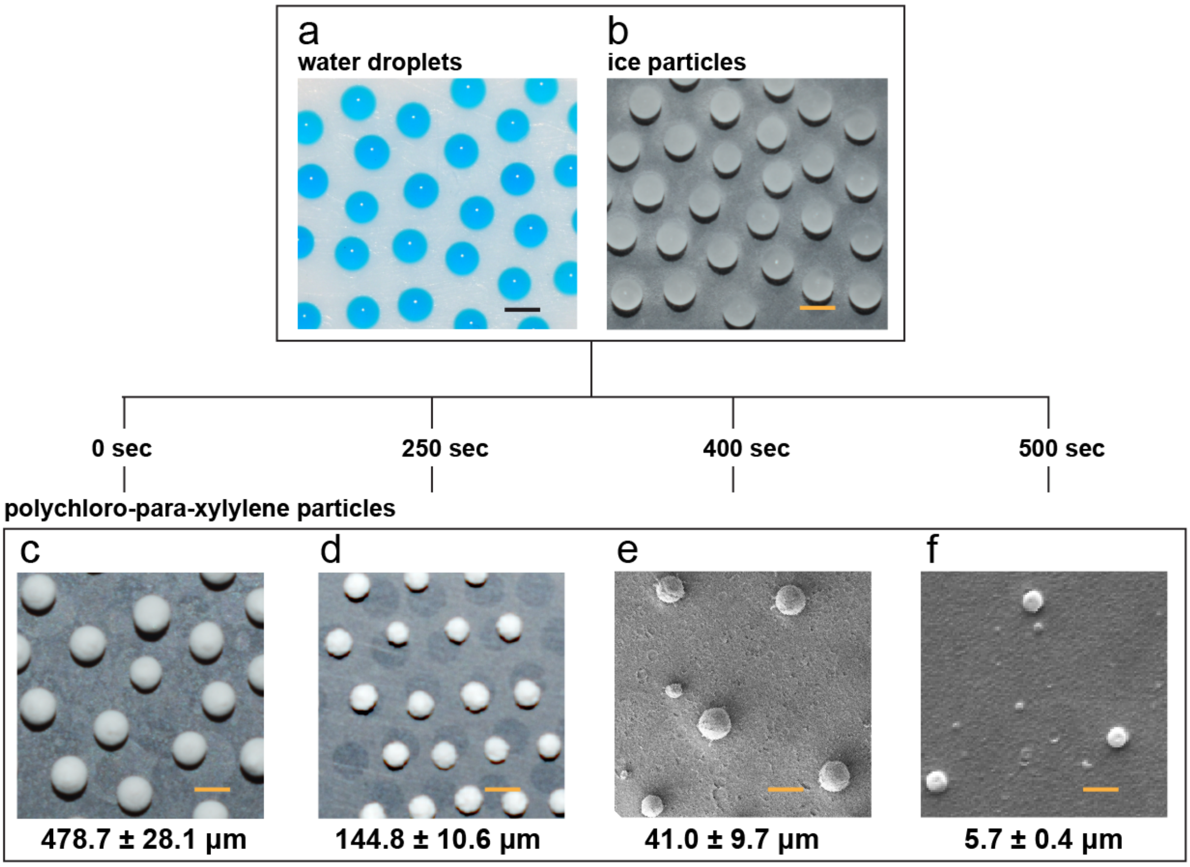

Supplementary Figure 1. Formation of (a) water droplets and (b) ice particles with uniform size (508.0 ± 6.5 µm) by using a pipette to transfer water liquids on a polytetrafluoroethylene (PTFE) surface. Scale bars: 500 µm. The ice particles were used as templates to produce polychloro-*para*-xylylene particles with controllable size including (c) 478.7 ± 28.1 µm (scale bar: 500 µm), (d) 144.8 ± 10.6 µm (scale bar: 200 µm), (e) 41.0 ± 9.7 µm (scale bar: 50 µm), and (f) 5.7 ± 0.4 µm (scale bar: 10 µm), based on varying the parameter of sublimation time during the stage-wise sublimation/deposition process. The samples were prepared separately.

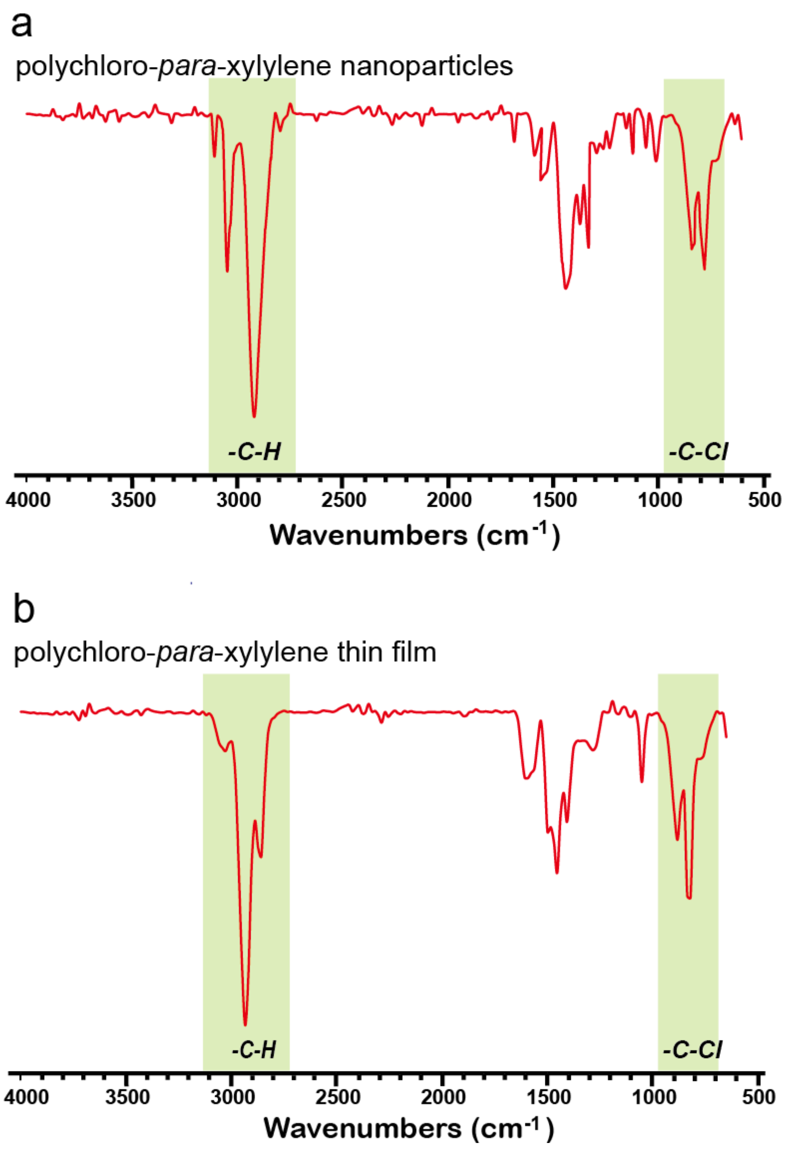

Supplementary Figure 2. The FT-IR spectrum of (a) the porous polychloro-*para*-xylylene particles was consistent with the spectrum from (b) a polychloro-*para*-xylylene thin film prepared using the conventional CVD process on a stationary substrate. Characteristic band absorptions indicated a *-C-Cl* peak from 709 to 925 cm^{-1} and *-C-H* in the range from 2793 to 2977 cm^{-1}.

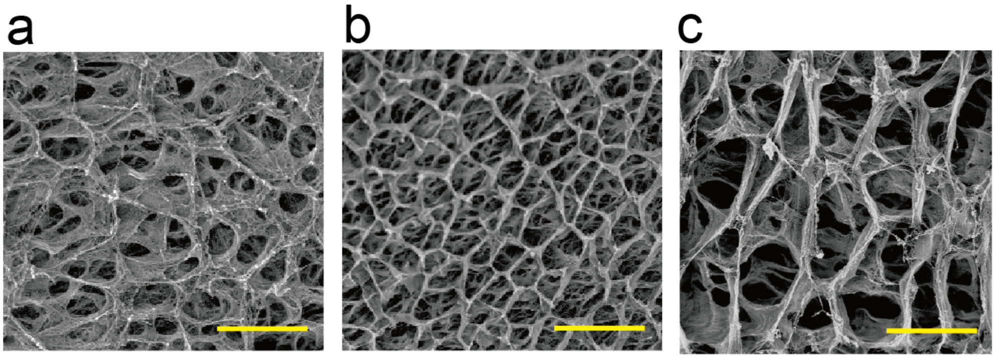

Supplementary Figure 3. Additional SEM images of the pore structures within the polychloro-*para*-xylylene particles. The pore size and void space were positively correlated to the sublimation rate of the ice particle template during the sublimation/deposition fabrication process. (a) Sublimating ice particles at -15 °C resulted in polymer particles with an approximately 277.1 ± 54.3 nm pore size, and (b) a void space of 45.0 ± 3.1 %; 4 °C resulted in 559.4 ± 94.8 nm and 61.7 ± 4.3 %, and (c) 25 °C resulted in 1260.5 ± 135.6 nm and 78.5 ± 6.2 %, respectively. The mean value ± S.D. was calculated from particles in six independent measurements. Scale bars: 1 μm.

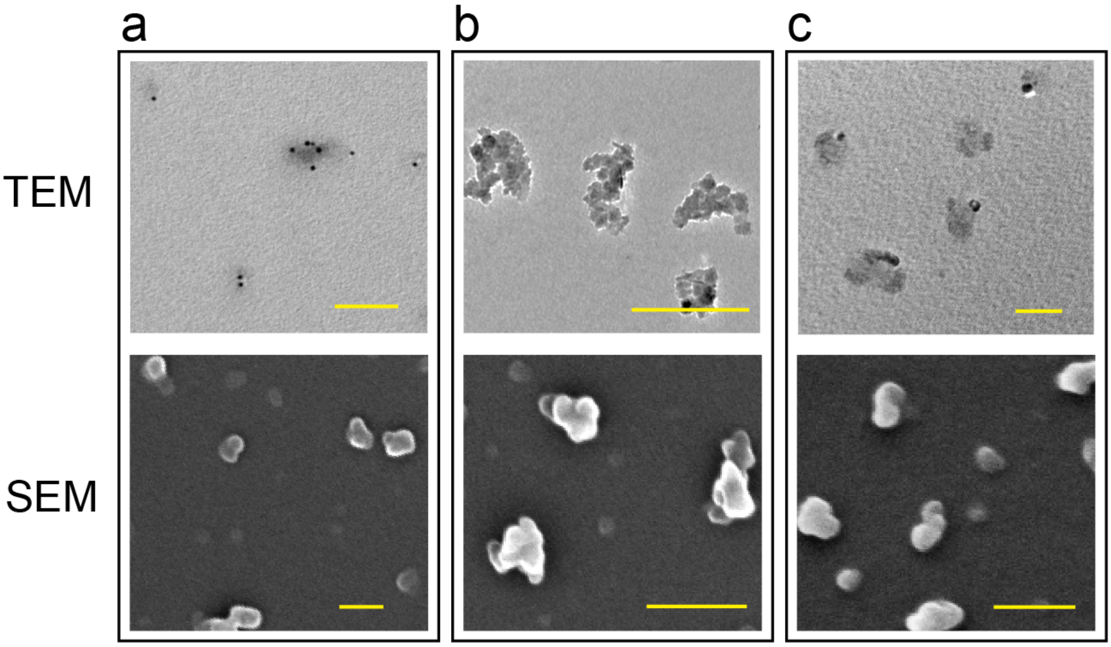

Supplementary Figure 4. TEM and SEM images of particle composites by using a multicomponent poly-*para*-xylylene copolymer to incorporate metals or oxides including (a) gold nanoparticles (scale bars: 100 nm), (b) Fe_3O_4 nanoparticles (scale bars: 200 nm), and (c) silver nanoparticles (scale bars: 100 nm), during the sublimation/deposition fabrication process.
